# The Role of IVC Filters in the Management of Acute Pulmonary Embolism

**DOI:** 10.3390/jcm13051494

**Published:** 2024-03-05

**Authors:** Samer Asmar, George Michael, Vincent Gallo, Mitchell D. Weinberg

**Affiliations:** 1 Division of Cardiology, Department of Internal Medicine, Staten Island University Hospital, Staten Island, NY 10305, USA; mweinberg4@northwell.edu; 2 Division of Vascular & Interventional Radiology, Department of Radiology, Staten Island University Hospital—Northwell Health, Staten Island, NY 10305, USA; gmichael@northwell.edu (G.M.); vgallo2@northwell.edu (V.G.)

**Keywords:** pulmonary embolism, IVC filter, high-risk PE, intermediate-risk PE

## Abstract

Venous thromboembolism (VTE), comprising deep venous thrombosis (DVT) and pulmonary embolism (PE), is a prevalent cardiovascular condition, ranking third globally after myocardial infarction and stroke. The risk of VTE rises with age, posing a growing concern in aging populations. Acute PE, with its high morbidity and mortality, emphasizes the need for early diagnosis and intervention. This review explores prognostic factors for acute PE, categorizing it into low-risk, intermediate-risk, and high-risk based on hemodynamic stability and right ventricular strain. Timely classification is crucial for triage and treatment decisions. In the contemporary landscape, low-risk PE patients are often treated with Direct Oral Anticoagulants (DOACS) and rapidly discharged for outpatient follow-up. Intermediate- and high-risk patients may require advanced therapies, such as systemic thrombolysis, catheter-directed thrombolysis, mechanical thrombectomy, and IVC filter placement. The latter, particularly IVC filters, has witnessed increased usage, with evolving types like retrievable and convertible filters. However, concerns arise regarding complications and the need for timely retrieval. This review delves into the role of IVC filters in acute PE management, addressing their indications, types, complications, and retrieval considerations. The ongoing debate surrounding IVC filter use, especially in patients with less conventional indications, reflects the need for further research and data. Despite complications, recent studies suggest that clinically significant issues are rare, sparking discussions on the appropriate and safe utilization of IVC filters in select PE cases. The review concludes by highlighting current trends, gaps in knowledge, and potential avenues for advancing the role of IVC filters in future acute PE management.

## 1. Introduction

Venous thromboembolism (VTE), including deep venous thrombosis (DVT) and pulmonary embolism (PE), ranks as the third most common cardiovascular ailment after myocardial infarction and stroke [1]. The risk of VTE doubles with each decade beyond age 40 and is thus becoming increasingly important in countries with aging populations [2]. Acute PE carries a high morbidity and mortality and, as such, necessitates an emphasis on early diagnosis and treatment [3]. A variety of rapidly expanding clinical, serologic, and imaging-based factors help prognostication of patients with acute PE [4]. PE can be categorized into low-risk, intermediate-risk (or sub-massive), and high-risk based on hemodynamic stability and the presence of right ventricular strain. Hemodynamically unstable patients are identified by a systolic blood pressure (SBP) below 90 mmHg, a drop in SBP of 40 mmHg or more from baseline, or the need for inotropes or vasopressors. Among hemodynamically stable patients, PE is considered low-risk if there is no evidence of right heart strain, and intermediate-risk in the presence of right heart strain identified through imaging, cardiac biomarker, and/or echocardiographic changes. PE is classified as high-risk when there is hemodynamic instability [5]. Appropriately categorizing a patient with PE as high-risk, intermediate-risk, or low-risk at the time of their presentation is uniquely impactful for early triage and treatment decisions.

In the modern era, low-risk patients are typically treated with Direct Oral Anticoagulants (DOACS) and, in appropriate settings, rapidly discharged for outpatient follow-up. Intermediate- and high-risk PE patients require parenteral anticoagulation and are considered for more advanced therapies in appropriate clinical scenarios, specifically those patients with high-risk PE and those intermediate-risk PE patients with certain high-risk features [6]. Such therapies include systemic thrombolysis, catheter-directed thrombolysis, mechanical thrombectomy, surgical thrombectomy, ECMO (extracorporeal membrane oxygenation), and inferior vena cava (IVC) filter placement. The data around the appropriate use of such therapies are still being generated via a plethora of ongoing trials, making this topic of advanced therapies for PE a topic of interest worldwide. Somewhat less glamorous but still important is the role of IVC interrupting devices in patients with PE. While IVC filters do not directly address acute VTE, they aim to prevent acute larger PE when the source of embolism originates in the venous system distal to the filter implantation site. However, smaller clots can still flow through the larger spaces of the filtering structure and is an acceptable trade-off between the filter catching all clots while maintaining IVC patency. This introduces an important distinction that should be made regarding filter implantation in patients with acute PE and confirmed DVT and in those whose imaging did not show the presence of thrombi in proximal or distal veins. Although the process is straightforward, it entails transporting the patient to the catheterization laboratory, puncturing a major vein (jugular, femoral, or arm vein), temporary immobilization of the patient, fluoroscopy, applying pressure to the vascular access site post-procedure, and, occasionally, readmission to remove the filter. It should be acknowledged that the procedure presents both organizational and economic challenges and may subject the patient to additional discomfort and anxiety. Indications for IVC filter placement in acute PE may be grouped into classic, well-accepted indications for use, and “extended”, less uniformly accepted indications. IVC filter use truly hinges on risk assessment and Eized considerations, especially when the indication for placement is less well-accepted [7]. Classic indications include patients with documented acute PE possessing absolute contraindications to anticoagulation or patients with high-risk PE considered to be at risk of death despite anticoagulation, or patients with VTE and a complication of anticoagulation. Far more controversial is the use of IVC filters in patients who have medical comorbidities that are thought to limit their cardiopulmonary reserve [8,9], a decision based on the concern that another PE in such a patient could be fatal, and thus, IVC filter placement is indicated in the absence of a conventional indication.

IVC filters provide protection from life-threatening PE in the early period, but over time long-term risks and filter complications increase. Over the time-period of an IVC filter, an initial favorable risk/benefit ratio changes to be less benefit and more risk at which point the filter should be removed [10,11]. The Society of Interventional Radiology (SIR) has established defined complications and acceptable thresholds for IVC filters. Supplementary Table S1 summarizes the most reported complications by clinicians, and Supplementary Figure S1 shows radiographic images of such complications. This has prompted considerable focus within the vascular and interventional radiology communities on prompt removal of retrievable filters within weeks to months, a window that varies by retrievable filter type. In early generation retrievable IVC filters, some devices had recommendations about the window of opportunity for removal, but current generation devices and even many early generation IVC filters can now be safely removed with interventional radiology techniques [12,13]. Importantly, Johnson et al.’s findings from The Predicting the Safety and Effectiveness of IVC Filters (PRESERVE) trial showed that while IVC filter complications do occur, those that are clinically significant are rare with currently available filters [14]. While both appropriate and inappropriate IVC filter use are associated with risk, a role remains for IVC filter use in select patients with PE. Selecting such patients, however, is limited by the paucity of high-quality data in the field. This review aims to explore the available data on IVC filters in acute PE management, discuss current trends impacting decision-making, and highlight opportunities for advancements that may potentially enhance the role of IVC filters in the future management of acute PE.

## 2. Historical Overview and Currently Available IVC Filters

**Figure 1:**
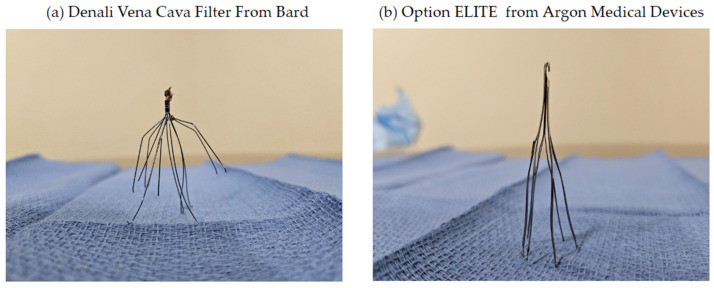
Different types of IVC filters available.

From a historic perspective, surgery was used to place clips around the IVC or sutures to segment the IVC before IVC filters were applied by Lazar Greenfield. This filter was placed using either a cutdown of the jugular or femoral vein and used a 24 Fr sheath. Interventional radiologists adopted the technique using percutaneous access (Dr. Dorfman, Brown University) [7]. Since then, IVC filter delivery systems have been greatly reduced in size making insertion much easier, and the use of IVC filters in the United States has steadily increased since the introduction of the Greenfield filter in 1972 [7]. In 2003, the FDA approved modifications to three permanent filters, enabling percutaneous retrieval [7]. Currently, IVC filters are categorized as permanent or optional, with the latter including temporary, retrievable, and convertible types. Retrievable IVC filters are sometimes referred to as temporary filters, even though they are FDA-cleared for both permanent and temporary placement, while temporary filters are specifically designed to be implanted only on a temporary basis and cannot be used permanently. Temporary filters are designed for short-term use and are suspended by catheters or wires. Convertible filters may be transformed into stents when IVC filtration is no longer needed. Retrievable filters possess tethering hooks for anchoring, like many permanent devices, but also possess a hook for later retrieval [10,11]. The filters used in the PRESERVE trial were ALN (ALN ± hook), Argon (Option Elite), B. Braun (LP, VenaTech Convertible), CR Bard (Denali), Cook (Gunther Tulip), Cordis (OptEase, TrapEase), and Philips Volcano (Crux) [14]. Figure 1 depicts some of the different types of IVC filters.

## 3. The Evolution in the Role of IVC Filters in the Management of Acute PE: The Two Eras

Our understanding of IVC filter use is best perceived upon the timeline of advancing PE therapeutics. Our initial understanding of the use of IVC filters in acute PE, until approximately 2010, was driven by a few data sets of limited size established when PE therapeutics were effectively simple. The introduction of PE multi-disciplinary care teams and the rapid advances in PE catheter-based technologies together drove a more aggressive strategy toward treating both high-risk and intermediate-risk PE. Systemic lysis at varying doses, catheter-based intervention, surgical thrombectomy, and ECMO use have all become more common as clinical understanding and related therapeutic strategies have evolved [10]. Data sets generated during this latter period are, of course, different than those generated in years prior to these advances. Thus, we will examine IVC filter data generated in these two very different eras: the early PE era and the current era.

### 3.1. The Early Era of PE Care: Marked by Registries, Trials, and In-Hospital Data

#### 3.1.1. Registries Demonstrate Limited PE Therapies during the Early Era

The landmark PE trial of the early era was The International Cooperative Pulmonary Embolism Registry (ICOPER). ICOPER was a large-scale, multicenter, prospective registry dedicated to the study of acute PE conducted in the mid-1990s and enrolled 2454 patients with acute PE across 52 institutions in North America and Europe. The study concluded that PE continues to be a significant clinical challenge with a high mortality rate (12 to 14% 90-day mortality) and provided valuable insights for the planning of future trials involving high-risk PE patients [15]. The Emergency Medicine Pulmonary Embolism in the Real-World Registry (EMPEROR) was a registry comprising consecutive emergency department (ED) outpatients diagnosed with acute PE over a 26-month period from 2006 to 2008 across 22 hospitals in the United States. 1880 patients with confirmed acute PE were enrolled, and the study concluded that these patients have high functional status and 1% mortality. It also highlighted that the management of acute PE patients in the ED with anticoagulation is poorly standardized and encouraged more research to improve outcomes in these patients [16]. Results from the EMPEROR registry and ICOPER also provided important information on IVC filter use. In the ICOPER, none of the 11 (10.1%) patients who received an IVC filter developed recurrent PE within 90 days, and 10 (90.9%) survived at least 90 days. They showed that IVC filters were associated with a reduction in 90-day mortality (hazard ratio, 0.12; 95% CI, 0.02 to 0.85) [14]. In EMPEROR, 9 out of 58 patients with massive PE (defined as SBP < 90 mm Hg) received IVC filters, and 273 out of 1817 patients with non-massive PE (defined as SBP ≥ 90 mm Hg) received IVC filters. Unfortunately, no sub-analysis was performed to look at whether the use of IVC filters improves mortality or not [16]. Reports from both the EMPEROR registry and ICOPER indicated low rates of systemic thrombolysis administration in patients with high-risk PE. In ICOPER, 33 patients (30.5%) underwent thrombolysis, 1 (0.9%) underwent catheter-directed therapy, and 3 (2.7%) had surgical embolectomy [14]. In EMPEROR, 7 patients (12.1%) underwent thrombolysis, none underwent catheter-directed therapy, and 2 (3.4%) had surgical embolectomy [16]. This therapeutic style is in stark contrast to a recent analysis which demonstrated that over 70% of patients with high-risk PE received advanced therapies, including systemic thrombolysis, which was the most common but still less than half, and a variety of other advanced therapies including catheter-directed thrombolysis and surgical embolectomy [17]. 

#### 3.1.2. The Trials

Randomized controlled trials (RCT) evaluating IVC filter use from the early era are few and limited by sample size. Perhaps the most important was the Prévention du Risque d’Embolie Pulmonaire par Interruption Cave II (PREPIC II) investigation. In the PREPIC II RCT, 200 stable patients with PE, along with DVT or superficial venous thrombosis and at least one additional high-risk criterion, received a retrievable IVC filter along with anticoagulation, while 199 patients received anticoagulation alone. Results at three and six months post-filter-insertion revealed comparable rates of recurrent PE, fatal PE, and all-cause mortality in those who received an IVC filter compared with those who did not [18]. The PREPIC II was limited in terms of assessment of utility for IVC filters in patients receiving anticoagulation; it helped further solidify the general approach to avoid filters in patients that can receive anticoagulation but did not help in understanding appropriateness in patients with a contraindication to anticoagulation, the group where filters are most commonly utilized. Other limitations included the exclusion of unstable patients and the absence of subgroup analysis given the small sample size. The PRESERVE trial is a large-scale, multi-specialty, nonrandomized prospective clinical study at 54 sites in the United States that enrolled 1429 participants who received IVC filters between 2015 and 2019. Patients were evaluated at baseline and followed up, even if the filter was removed, to determine the safety and effectiveness of vena cava filters. The PRESERVE trial by Johnson et al. affirmed the safety of IVC filters but faced challenges in claiming effectiveness. Limitations included the absence of a control group, inclusion of patients with anticoagulation history, and a lack of routine imaging for recurrent VTE assessment. The study’s design impedes a direct comparison between IVC filter placement and medical management, hindering a clear assertion of the intervention’s effectiveness [14,19]. Bikdeli et al. conducted a systematic review and meta-analysis that included six RCT and five prospective observational studies to further evaluate the safety and efficacy of IVC filters versus none in 4204 patients at risk of PE. They concluded that IVC filters reduced the risk of subsequent PE (odds ratio, 0.50; 95% CI, 0.33 to 0.75), increased the risk for DVT (odds ratio, 1.70; 95% CI, 1.17 to 2.48), and had no significant effect on neither PE-related mortality (odds ratio, 0.51; 95% CI, 0.25 to 1.05) nor overall mortality (odds ratio, 0.91; 95% CI, 0.70 to 1.19). However, on post hoc analysis of three studies whose patients had contraindications to anticoagulation and recurrent PE despite adequate anticoagulation, the nonsignificant reduction in PE-related mortality reached statistical significance (odds ratio, 0.47; 95% CI, 0.21 to 1.04) [1]. This study subgroup is most reflective of current guideline-recommended indications guiding IVC filter placement. Important limitations disclosed by the authors included the lack of a control procedure which may potentially bias the results of the individual studies and thereby contribute to pooled estimates, a likely underestimation of the rates of IVC-filter-related complications, and the exclusion of all retrospective studies.

#### 3.1.3. In-Hospital Data

Registry data from the early era suggested the opposite, that IVC filters might be useful in patients with acute PE and certain high-risk features. Stein et al. conducted an analysis of the 1979 to 1999 National Hospital Discharge Survey (NHDS) database and revealed a consistent linear increase in the percentage of acute PE patients who underwent IVC filter insertion over a 21-year observation period [20]. This increase in the utilization of IVC filters in the management of acute PE provided a rich data set to potentially answer questions regarding the clinical utility of IVC filters in acute PE. Stein et al., in a review article, assessed the utility of IVC filters in stable patients with acute PE [21]. Results suggested that a variety of patient subsets, such as those undergoing pulmonary embolectomy, receiving thrombolytic therapy, experiencing recurrent PE while on treatment, hospitalized with solid malignant tumors (particularly if aged > 60 years), hospitalized with chronic obstructive pulmonary disease (COPD) (especially if aged > 50 years), and affected by PE when elderly (aged > 80 years), all exhibited reduced mortality with the addition of an IVC filter [21]. 

### 3.2. The Second Era: Marked by Novel Changes

**Figure 2:**
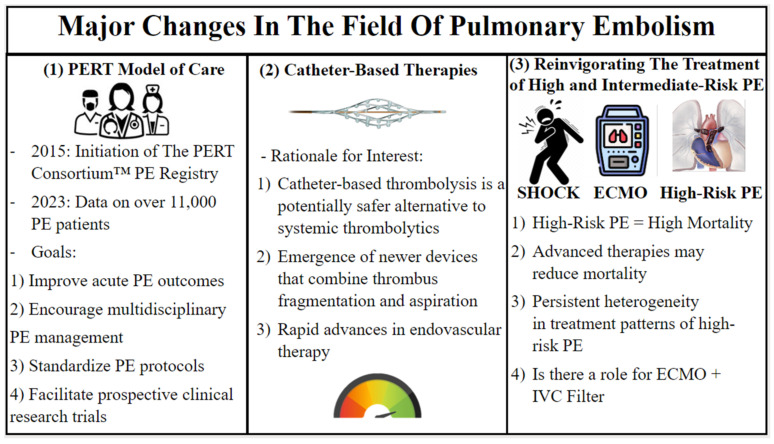
Major changes in the field of pulmonary embolism.

The second era is marked by a set of clinical and device-related advances in the care of patients with acute PE. These advances in interventional tools, PE risk stratification, lytic dosing strategy, IVC filter technology, and shock management have reinvigorated the diagnosis and treatment of high- and intermediate-risk PE and prompted a reevaluation of IVC filter usage in PE patients. Another major change in PE was the development of safer and more reliable retrievable IVC filters. Before this, a patient with VTE had a permanent device inserted which may indwell for decades. While the development of the filters perhaps lowered the threshold to apply them in VTE, the difficulty is identifying the subset(s) of patient who will benefit the most from IVC filtration (Figure 2).

Secemsky et al. analyzed 630 patients with high- and intermediate-risk PE and found that advanced therapies were independently associated with 61% reduction in mortality despite major bleeding events. Of these patients, 37.9% received advanced therapy distributed as follows: IVC filter (20.7%), systemic thrombolysis (4.7%), catheter-directed thrombolysis (13.9%), endovascular suction embolectomy (0.9%), surgical embolectomy (4.4%), or ECMO (2.1%) [22]. Advanced therapies are increasingly being looked at and utilized, and further investigation is needed to determine their optimal use.

## 4. PERT and Other Societal Interest in IVC-Related Research

The PE Response Team (PERT) Consortium™ PE Registry is a contemporary multicenter registry designed to adapt to the evolving healthcare landscape, emphasizing a value-based system that prioritizes measurable aspects of quality, cost, and patient experience. This registry established and promoted the multidisciplinary PERT model of care delivery. Early publications from the PERT Consortium™ PE Registry included studies on PE mortality risk scores, risk stratification, and management practices among PE experts [10,23]. Variability in practice patterns was observed among participating centers, with advanced therapy implementation ranging from 16% to 46%, and 30-day mortality varying from 9% to 44% [10]. The diverse practices observed in the studies emphasize the urgency of establishing guidelines that promote optimal care, reduce variability, and improve overall quality in the management of acute PE. Driven by the PERT consortium, a renewed focus and societal interest on PE-related research occurred, and novel studies emerged discussing the role of IVC filters in acute PE. The American College of Chest Physicians, the American Heart Association, and the SIR have published guidelines for IVC filter insertion. The guidelines from the American College of Chest Physicians were perhaps the most conservative when it comes to insertion of IVC filters. The Eastern Association for the Surgery of Trauma also has guidelines for IVC filter placement in trauma patients [9]. However, the multiplicity of groups and varying recommendations makes it confusing for many as to when and who should get IVC filters.

## 5. Exploring the Role of IVC Filters in Diverse Patient Populations with Acute PE

### 5.1. High-Risk and Intermediate-Risk PE

No randomized controlled trials have been conducted to assess the efficacy of thrombolytic therapy, pulmonary embolectomy, or IVC filters in patients experiencing high-risk PE, characterized by shock or the need for ventilator support. Among intermediate-risk PERT-assessed patients in the registry, 32% received catheter-directed therapies, and 7% had an IVC filter placed. For high-risk patients, 37% underwent catheter-directed therapies, 25% received tissue plasminogen activator, 12% had an IVC filter implanted, and 14% were placed on ECMO [10]. These findings derived from the PERT consortium guided the design of observational studies to address the indications of IVC filters in the management of acute PE. Important inclusion/exclusion criteria include the patient’s hemodynamic status (stable or unstable) at the time of acute PE and whether advanced therapies (systemic thrombolysis, catheter-directed thrombolysis, endovascular suction embolectomy, surgical embolectomy, or ECMO) were used. Elderly patients and patients with recurrent PE are unique populations with separate indications for IVC filter placement. The subsequent discussion provides a review of the studies supporting the use of IVC filters in different patients with acute PE. Despite the absence of such trials, numerous investigations have explored these treatments based on retrospective cohort studies utilizing administrative data from large government and commercial databases.

#### 5.1.1. Patients Receiving Thrombolytic Therapy

A variety of non-randomized analyses have suggested that IVC filters may improve mortality when used in intermediate- and high-risk PE patients receiving lytic therapy. To ascertain the role of IVC filters in acute PE management, Stein et al., using administrative data from large government and commercial databases, demonstrated that outcomes of thrombolytic therapy were significantly enhanced when IVC filters were incorporated. Specifically, IVC filters demonstrated a reduction in in-hospital all-cause mortality not only when used alongside anticoagulants alone (mortality IVC filter 32% vs. mortality no IVC filter 51%, *p* < 0.0001) or in pulmonary embolectomy (mortality IVC filter 24% vs. mortality no IVC filter 58%, *p* < 0.0001), but also in conjunction with thrombolytic therapy (mortality IVC filter 8% vs. mortality no IVC filter 18%, *p* < 0.0001) across all age groups in individuals with high-risk PE. The effectiveness of IVC filters in reducing mortality was particularly notable when inserted on the day of admission or the following day, during the period when the patient is most fragile. This suggests that the optimal treatment for patients with high-risk PE involves the combination of thrombolytic therapy and IVC filter insertion in the early stage when the patient is actively unstable. The authors conclude that this combined treatment approach is recommended for all high-risk PE patients, regardless of age [24]. In a single-center prospective study, Secemsky et al. evaluated outcomes in acute PE patients and noted that mortality was highest during the index hospitalization for high-risk PE patients, a risk that dissipated at the time of discharge. However, in the patients with intermediate-risk PE, the mortality risk persisted beyond the time of discharge [22]. Notably, advanced therapies, including IVC filters, were commonly used in this population and demonstrated an independent association with lower mortality (hazard ratio, 0.39, 95% CI, 0.20 to 0.76; *p* < 0.01), a finding consistent with other studies [22,25]. A subsequent analysis by Stein et al. reinforced the importance of early IVC filter insertion and demonstrated that in-hospital all-cause mortality appeared to be reduced with IVC filter placement (mortality IVC filter 19.4% vs. mortality no IVC filter 40.8%, *p* < 0.0001) only when the filter was inserted on the first (mortality IVC filter 21.4% vs. mortality no IVC filter 40.8%, *p* = 0.017) or second day of admission (mortality IVC filter 14.8% vs. mortality no IVC filter 29.2%, *p* = 0.023). This outcome benefit was independent of thrombolytic therapy administration [26]. Interestingly, a separate study demonstrated that advanced age should not be a limiting factor when considering an IVC filter in high-risk patients with PE [27]. Combining these more recent studies with the results of NIS database analyses from the early era makes for a convincing argument for IVC filter placement in high-risk and intermediate-risk PE patients.

#### 5.1.2. Patients Receiving Pulmonary Embolectomy

The American College of Chest Physicians recommends surgical pulmonary embolectomy in cases where patients have contraindications to thrombolytic therapy, have experienced failed thrombolysis or catheter-assisted embolectomy, or are in a state of shock that is likely to lead to death before the effects of thrombolysis can take place, provided that surgical expertise and resources are available [28]. Notably, three retrospective cohort studies, spanning different time periods and utilizing various databases such as the Nationwide Inpatient Sample (NIS) (1999–2008) (mortality IVC filter 25% vs. mortality no IVC filter 58%, *p* < 0.0001), the Premier Healthcare Database (2010–2014) (mortality IVC filter 5.9% vs. mortality no IVC filter 44%, *p* = 0.01), and the NIS (2009–2014) (mortality IVC filter 18.1% vs. mortality no IVC filter 50.1%, *p* < 0.0001), demonstrated a lower mortality associated with the use of IVC filters in high-risk PE patients who underwent pulmonary embolectomy [29,30,31]. 

Stein et al. conducted a retrospective analysis using data from the 2010–2014 Premier Healthcare Database to evaluate the impact of IVC filters on mortality in patients with high-risk PE and those who underwent pulmonary embolectomy [30]. Their findings indicated that patients with high-risk PE who received an IVC filter exhibited lower in-hospital all-cause mortality (mortality IVC filter 23% vs. mortality no IVC filter 45%, *p* < 0.0001) and lower 3-month all-cause mortality (mortality IVC filter 25% vs. mortality no IVC filter 45%, *p* < 0.0001) compared to those without an IVC filter. This reduction in mortality was observed in patients receiving thrombolytic therapy, undergoing pulmonary embolectomy, and those receiving neither. Moreover, mortality attributable to PE at both in-hospital and 3-month intervals was also lower in patients who received an IVC filter in each subgroup [30]. 

#### 5.1.3. Patients Receiving ECMO or Surgical Thrombectomy

Patients with high- and intermediate-risk PE may be excellent candidates for veno-arterial (VA)-ECMO and some potential indications for ECMO include patients with absolute contraindications to thrombolysis, persistent instability despite thrombolysis (lytic failure), and the stabilization of a patient prior to intubation. Future potential roles in the management of high- and intermediate-risk PE patients may include its role as a temporizing bridging therapy until anticoagulation efficacy, controlled thrombolysis, or definitive interventional therapy is performed [32]. Liu et al. performed a 2018–2021 single-center retrospective review of a prospectively maintained registry and included nine patients with high- and intermediate-risk PE who underwent VA-ECMO for initial hemodynamic stabilization, with or without percutaneous mechanical thrombectomy. Only two of the nine patients (22.2%) received IVC filters. They concluded that an ECMO-first strategy in these patients was safe and efficacious. Specifically, they consider VA-ECMO as a feasible option for initial stabilization, serving as a bridge to therapy, particularly in cases where surgery is not feasible for high-risk PE. To date, obtaining high-level evidence is challenging due to the rarity of situations requiring VA-ECMO in acute PE and the restricted availability of ECMO to specialized centers [33]. In recent years, surgical thrombectomy, once considered risky and generally ineffective, has experienced a resurgence. The current mortality rate for the procedure is approximately 10%, deemed acceptable in specific high-risk cases. Potential indications for surgical thrombectomy include high-risk PE in patients with absolute contraindications to thrombolysis or cases of thrombolytic failure. Currently, there is no high-level evidence comparing surgical thrombectomy to interventional radiology clot extraction but advances in catheter embolectomy, such as the Inari Flowtriever system, may offer superior outcomes in many cases, but further study is needed [34]. Informal discussions between many interventional radiology groups in the San Diego area, where utilization of these suction thrombectomy devices is increasingly being utilized, suggests improved patient hemodynamics, successful outcomes, and faster hospital discharges. However, the ideal patients who are the best candidates for such, more aggressive, therapies remain a topic of debate. While it is difficult to give a strong recommendation to place an IVC filter in patients on ECMO, case reports suggest placing one in high-risk PE patients with idiopathic hypercoagulability and residual thrombus despite thrombolytic therapy, regardless of the use of percutaneous mechanical thrombectomy. Implanting the IVC filter should be performed in tandem with ECMO decannulation to avoid potentially lethal complications [33,35]. 

### 5.2. Stable Acute PE

Stein et al. utilized the 2010–2014 Premier Healthcare Database to demonstrate that among stable patients with acute PE who underwent thrombolytic therapy, those who additionally received an IVC filter experienced lower in-hospital all-cause mortality (mortality IVC filter 5.2% vs. mortality no IVC filter 16.1%, *p* < 0.0001). This reduction in in-hospital mortality was observed across all age groups from 31 years and older for individuals who received an IVC filter in conjunction with thrombolytic therapy [36]. Results from the 2009–2014 NIS database showed similar benefits in stable acute PE patients who underwent pulmonary embolectomy and received IVC filters (mortality IVC filter 4.1% vs. mortality no IVC filter 27%, *p* < 0.0001), specifically if filters were inserted within the first 4 or 5 days following embolectomy [31]. The older literature derived from the 1999–2014 NIS database showed that there is no substantial evidence supporting a clinically meaningful reduction in mortality with IVC filters in stable patients, unless they are aged over 80 years [27]. Despite these positive outcomes, the studies highlighted a concerning trend; the proportion of unstable patients receiving IVC filters is decreasing, with the largest number of filters continuing to be inserted in stable patients with acute PE [26,37]. 

### 5.3. Elderly Patients with Acute PE

In an investigation focusing on elderly patients (≥65 years old) with stable acute PE who did not receive thrombolytic therapy, a national cohort study of Medicare beneficiaries revealed that the use of IVC filters did not result in lower all-cause mortality at 30 days [38]. However, in a subsequent assessment by Stein et al., utilizing more recent data from the NIS, they concluded that in very elderly stable patients (aged >80 years) with a primary or secondary diagnosis of acute PE, with (mortality IVC filter 6.1% vs. mortality no IVC filter 10.5%, *p* < 0.0001) or without (mortality IVC filter 3.3% vs. mortality no IVC filter 6.3%, *p* < 0.0001) comorbid conditions, the use of IVC filters led to a reduction in mortality [27]. Furthermore, in another study by Stein et al., stable patients with PE and heart failure (HF) who were aged >80 years exhibited reduced in-hospital all-cause mortality (mortality IVC filter 4.1% vs. mortality no IVC filter 6.8%, *p* = 0.0012) when IVC filters were employed [39]. Another special population that may benefit from IVC filters in acute PE includes stable patients with PE and solid malignant tumors, specifically those older than 60 years. This subgroup demonstrated lower in-hospital all-cause mortality (mortality IVC filter 7.4% vs. mortality no IVC filter 11.2%, *p* < 0.0001) and lower 3-month mortality (mortality IVC filter 15.1% vs. mortality no IVC filter 17.4%, *p* < 0.0001) compared to those who did not receive an IVC filter [40]. 

### 5.4. Patients Experiencing Recurrent PE

In 2016, a cohort study involving patients from the Registro Informatizado de la Enfermedad Tromboembolica (RIETE registry) demonstrated a lower mortality (mortality IVC filter 2.1% vs. mortality no IVC filter 25.3%, *p* = 0.02) associated with the use of IVC filters in patients experiencing recurrent PE while on anticoagulant therapy [41]. Additionally, Stein et al. conducted a retrospective cohort study spanning six years, using administrative data from the Premier Healthcare Database. Their findings concluded that patients with PE who suffered a recurrent PE within the first three months after an index PE exhibited reduced mortality (mortality IVC filter 3.0% vs. mortality no IVC filter 39.3%, *p* < 0.0001) if they received an IVC filter at the time of recurrence. This reduction in mortality was observed in stable patients who did not receive thrombolytic therapy or undergo pulmonary embolectomy (mortality IVC filter 2.6% vs. mortality no IVC filter 42.6%, *p* < 0.0001). The study emphasized the importance of IVC filters in reducing mortality in stable patients with recurrent PE, underscoring the risk of death associated with early recurrences [42]. The high mortality rates reported in these studies suggest that patients with recurrent PE, despite therapeutic anticoagulation, are at the highest risk of mortality and IVC filters should be used in these cases.

## 6. Classic and Extended Indications for IVC Filters in Acute PE: Expert Panel Recommendations

Kaufman et al. conducted a systematic review and identified a total of 34 studies that provided the evidence base for the guidelines guiding IVC filter placement. The expert panel consisted of renowned experts across various medical, surgical, and interventional societies who agreed on the following recommendations with respect to acute PE [8,9]. However, they conclude that the efficacy of IVC filters in acute PE remains debatable, necessitating personalized assessments considering risks and benefits. Table 1 summarizes some of the recognized and potential advantages of IVC filter placement. Classic indications involve documented acute PE with absolute anticoagulation contraindications or a massive PE posing a risk of death despite anticoagulation. Despite the limited availability of robust evidence, the consensus among experts suggests that individuals facing acute PE along with contraindications to anticoagulation should generally be considered for IVC filter placement. Important factors influencing this decision encompass the patient’s cardiopulmonary condition, hemodynamic response to PE, and the anticipated duration of contraindication to anticoagulation. In patients with massive PE, hemodynamic shock, and/or requiring ventilatory support, the panel deems that the potential benefits, including a likely reduction in in-hospital mortality from recurrent PE, justify the intervention in this specific and select patient population. The expert panel concluded that, in these cases, the benefits associated with IVC filter placement, including a reduction in short-term PE recurrence and potentially a decrease in mortality, outweigh the potential harms.

Extended indications encompass cases treated with thrombolysis or thrombectomy and acute PE in individuals with limited cardiopulmonary reserve. In patients experiencing acute PE and undergoing advanced therapies, the expert panel issues a recommendation with limited strength for IVC filter placement, particularly in those with unstable conditions such as hemodynamic shock, requiring ventilatory support, and/or limited cardiopulmonary reserve. This recommendation is grounded in low-quality retrospective observational studies. The panel deems that the potential benefits, including a likely reduction in in-hospital mortality from recurrent PE, justify the intervention in this specific and select patient population. The panel also introduced the importance of making a distinction regarding IVC filter implantation in patients with acute PE regardless of advanced therapies and confirmed DVT to those whose imaging did not reveal the presence of thrombi in the proximal or distal veins. The panel deems those patients with acute PE and documented iliocaval DVT or large, free-floating proximal DVT as candidates for IVC filter placement satisfying extended indications.

## 7. Devising Novel IVC Filters and Retrieval Programs

Technological advance in the current era of PE care has not been limited to the treatment of PE in isolation. Currently, there are continuous efforts to improve IVC filter devices and design effective retrieval programs to improve outcomes in acute PE patients. These advances were built on Johnson et al.’s findings from the ongoing PRESERVE trial in which major IVC filter manufacturers are actively involved [14].

(A) Novel IVC Filter Advance

The PRESERVE trial showed low complications with currently available filters: strut perforation greater than 5 mm was demonstrated in 31 of 201 (15.4%) filters, of which only 3 (0.2%) were considered clinically significant, and filter-related perioperative adverse events occurred in 7 of 1421 (0.5%) patients. On follow-up, VTE (none of which were fatal) occurred in 93 patients (6.5%), including DVT (80 events in 74 patients [5.2%]), PE (23 events in 23 patients [1.6%]), and/or caval thrombotic occlusions (15 events in 15 patients [1.1%]), and no PE occurred in patients following prophylactic placement [14]. IVC filter advances involve enhanced comprehension of filter-associated complications and novel filter manufacturing. The development of convertible and bio-convertible filters like Sentry and VenaTech models eliminates the need for additional removal procedures and addresses potential complications associated with indwelling filters, providing temporary protection against PE before retraction of the filter arms and stent-like incorporation into its surrounding vasculature [43]. Clinical trials, such as the investigational device exemption multicenter trial with a convertible IVC filter, report favorable conversion rates and low adverse effects [44]. Additionally, the FDA-approved triple-lumen central venous catheter with a deployable IVC filter provides protection in critically ill patients and must be removed before discharge to avoid long-term complications [45]. As filter technology continues to develop, so will the determination of their indications, safety, and efficacy.

(B) Rigorous IVC Filter Retrieval Programs and Their Efficacy

The future direction of IVC filter utilization in managing PE emphasizes the importance of increasing retrieval rates and avoiding potential long-term complications. Timely retrieval of IVC filters is an important quality metric which multidisciplinary PERT aims to improve by reducing unnecessary filter use, streamlining outpatient follow-up, and expediting filter removal. The time window for safe retrieval varies by filter subtype. The FDA issued a safety communication in 2014 based on reports of adverse events associated with IVC filters and recommended that implanting physicians consider removing the filter as soon as blood clots are no longer a risk for the patient. After this report, many operators became more serious about IVC filter removals, and referrals for IVC filters declined from previous levels [7]. Johnson et al.’s findings from the PRESERVE trial affirm the safety of IVC filters in contemporary medical practice. IVC filters were removed from 632 of 640 (98.8%) patients who underwent attempted removal, 620 (96.8%) of which were removed at first attempt. Only one patient died during attempted filter retrieval [14]. Similarly, De Gregorio et al. conducted a study in the Spanish multicenter real-life registry (REFiVeC), reporting a 94.15% global retrieval rate after adjustment with no major complications [46]. Efforts at improving retrieval rates should focus on physician accountability, emphasizing that practitioners should only place IVC filters when strong indications exist and that they are also responsible for removing them when they are no longer indicated. This can be better accomplished with well-designed and enforced follow-up plans at the time of placement. Improved expertise in advanced retrieval techniques is also crucial, with an acceptable target retrieval success rate of 95%. Lastly, standardizing rigorous protocols to enhance the retrieval rates and provide high-quality care for patients can only succeed when a multidisciplinary team-based approach is followed.

## 8. Conclusions

The percutaneous image-guided insertion of an IVC filter represents a crucial therapeutic option in the management of specific patients with acute PE. However, the strength of recommendations in various clinical scenarios is limited by the lack of high-quality data, which is a persistent challenge in the field. The multiplicity of guidelines across various medical disciplines adds to confusion and uncertainty about appropriate use of IVC filters. While it is crucial to approach the inference of lower mortality with IVC filters cautiously, given the reliance on comparative effectiveness research using national observational data, the prospect of conducting an RCT in these specific subcategories of acute PE patients appears remote. The decision on whether patients are better served by the proactive insertion of an IVC filter based on retrospective cohort studies or by withholding IVC filters until an RCT can be conducted requires careful consideration.

## Figures and Tables

**Table 1:** Recognized and potential advantages of IVC filters.

Recognized Advantages of IVC Filters	Potential Advantages of IVC Filters
Prevent acute larger PE when the source of embolism originates in the venous system distal to the filter implantation site	Development of newer and safer IVC Filters may lead to more utilization with better outcomes
Classic Indications: A Role In: (1) Patients with documented acute PE possessing absolute contraindications to anticoagulation (2) Patients with high-risk PE considered to be at risk of death despite anticoagulation (3) Patients with VTE and a complication of anticoagulation	Extended Indications: A Role In: (1) Patients treated with thrombolysis or thrombectomy (2) Acute PE in individuals with limited cardiopulmonary reserve (3) Acute PE and undergoing ECMO (4) Acute PE in unstable conditions such as hemodynamic shock and requiring ventilatory support (5) Patients with acute PE and documented iliocaval DVT or large, free-floating proximal DVT
Role in recurrent PE despite therapeutic anticoagulation	Role in elderly patients with acute PE

## Data Availability

Not applicable.

## Supplementary Materials

The following supporting information can be downloaded at: https://www.mdpi.com/article/10.3390/jcm13051494/s1, Table S1. IVC-Filter-Related Complications and Definitions; Figure S1. Radiographic Images of IVC-Filter-Related Complications.

## References

[1] Bikdeli B., Chatterjee S., Desai N.R., Kirtane A.J., Desai M.M., Bracken M.B., Spencer F.A., Monreal M., Goldhaber S.Z., Krumholz H.M. (2017). Inferior Vena Cava Filters to Prevent Pulmonary Embolism: Systematic Review and Meta-Analysis. J. Am. Coll. Cardiol..

[2] Raskob G.E., Angchaisuksiri P., Blanco A.N., Buller H., Gallus A., Hunt B.J., Hylek E.M., Kakkar A., Konstantinides S.V., Mccumber M. (2014). Thrombosis: A major contributor to global disease burden. Arterioscler. Thromb. Vasc. Biol..

[3] Aujesky D., Obrosky D.S., Stone R.A., Auble T.E., Perrier A., Cornuz J., Roy P.M., Fine M.J. (2006). A prediction rule to identify low-risk patients with pulmonary embolism. Arch. Intern. Med..

[4] Wood K.E. (2002). Major pulmonary embolism: Review of a pathophysiologic approach to the golden hour of hemodynamically significant pulmonary embolism. Chest.

[5] Balakrishna M.A., Reddi V., Belford P.M., Alvarez M., Jaber W.A., Zhao D.X., Vallabhajosyula S. (2022). Intermediate-Risk Pulmonary Embolism: A Review of Contemporary Diagnosis, Risk Stratification and Management. Medicina.

[6] Tapson V.F. (2008). Acute pulmonary embolism. N. Engl. J. Med..

[7] Muriel A., Jiménez D., Aujesky D., Bertoletti L., Decousus H., Laporte S., Mismetti P., Muñoz F.J., Yusen R., Monreal M. (2014). Survival effects of inferior vena cava filter in patients with acute symptomatic venous thromboembolism and a significant bleeding risk. J. Am. Coll. Cardiol..

[8] Kaufman J.A., Barnes G.D., Chaer R.A., Cuschieri J., Eberhardt R.T., Johnson M.S., Kuo W.T., Murin S., Patel S., Rajasekhar A. (2020). Society of Interventional Radiology Clinical Practice Guideline for Inferior Vena Cava Filters in the Treatment of Patients with Venous Thromboembolic Disease: Developed in collaboration with the American College of Cardiology, American College of Chest Physicians, American College of Surgeons Committee on Trauma, American Heart Association, Society for Vascular Surgery, and Society for Vascular Medicine. J. Vasc. Interv. Radiol..

[9] DeYoung E., Minocha J. (2016). Inferior Vena Cava Filters: Guidelines, Best Practice, and Expanding Indications. Semin. Interv. Radiol..

[10] Schultz J., Giordano N., Zheng H., Parry B.A., Barnes G.D., Heresi G.A., Jaber W., Wood T., Todoran T., Courtney D.M. (2019). EXPRESS: A Multidisciplinary Pulmonary Embolism Response Team (PERT)—Experience from a national multicenter consortium. Pulm Circ..

[11] Ortel T.L., Neumann I., Ageno W., Beyth R., Clark N.P., Cuker A., Hutten B.A., Jaff M.R., Manja V., Schulman S. (2020). American Society of Hematology 2020 guidelines for management of venous thromboembolism: Treatment of deep vein thrombosis and pulmonary embolism. Blood Adv..

[12] Duffett L., Carrier M. (2017). Inferior vena cava filters. J. Thromb. Haemost..

[13] Morales J.P., Li X., Irony T.Z., Ibrahim N.G., Moynahan M., Cavanaugh K.J. (2013). Decision analysis of retrievable inferior vena cava filters in patients without pulmonary embolism. J. Vasc. Surg. Venous Lymphat. Disord..

[14] Johnson M.S., Spies J.B., Scott K.T., Kato B.S., Mu X., Rectenwald J.E., White R.A., Lewandowski R.J., Khaja M.S., Zuckerman D.A. (2023). Predicting the Safety and Effectiveness of Inferior Vena Cava Filters (PRESERVE): Outcomes at 12 months. J. Vasc. Surg. Venous Lymphat. Disord..

[15] Goldhaber S.Z., Visani L., De Rosa M. (1999). Acute pulmonary embolism: Clinical outcomes in the International Cooperative Pulmonary Embolism Registry (ICOPER). Lancet.

[16] Pollack C.V., Schreiber D., Goldhaber S.Z., Slattery D., Fanikos J., O’Neil B.J., Thompson J.R., Hiestand B., Briese B.A., Pendleton R.C. (2011). Clinical characteristics, management, and outcomes of patients diagnosed with acute pulmonary embolism in the emergency department: Initial report of EMPEROR (Multicenter Emergency Medicine Pulmonary Embolism in the Real World Registry). J. Am. Coll. Cardiol..

[17] Lin B.W., Schreiber D.H., Liu G., Briese B., Hiestand B., Slattery D., Kline J.A., Goldhaber S.Z., Pollack C.V. (2012). Therapy and outcomes in massive pulmonary embolism from the Emergency Medicine Pulmonary Embolism in the Real World Registry. Am. J. Emerg. Med..

[18] Mismetti P., Laporte S., Pellerin O., Ennezat P.V., Couturaud F., Elias A., Falvo N., Meneveau N., Quere I., Roy P.M. (2015). Effect of a retrievable inferior vena cava filter plus anticoagulation vs anticoagulation alone on risk of recurrent pulmonary embolism: A randomized clinical trial. JAMA.

[19] Dawson D.L. (2023). PRESERVE trial confirms low risk for most inferior vena cava filters, but benefit remains uncertain. J. Vasc. Surg. Venous Lymphat. Disord..

[20] Stein P.D., Kayali F., Olson R.E. (2004). Twenty-one-year trends in the use of inferior vena cava filters. Arch. Intern. Med..

[21] Stein P.D., Matta F., Hughes M.J. (2019). Usefulness of Inferior Vena Cava Filters in Stable Patients with Acute Pulmonary Embolism. Am. J. Cardiol..

[22] Secemsky E., Chang Y., Jain C.C., Beckman J.A., Giri J., Jaff M.R., Rosenfield K., Rosovsky R., Kabrhel C., Weinberg I. (2018). Contemporary Management and Outcomes of Patients with Massive and Submassive Pulmonary Embolism. Am. J. Med..

[23] Kabrhel C., Rosovsky R., Channick R., Jaff M.R., Weinberg I., Sundt T., Dudzinski D.M., Rodriguez-Lopez J., Parry B.A., Harshbarger S. (2016). A Multidisciplinary Pulmonary Embolism Response Team: Initial 30-Month Experience with a Novel Approach to Delivery of Care to Patients with Submassive and Massive Pulmonary Embolism. Chest.

[24] Stein P.D., Dalen J.E., Matta F., Hughes M.J. (2019). Optimal Therapy for Unstable Pulmonary Embolism. Am. J. Med..

[25] Kobayashi T., Pugliese S., Sethi S.S., Parikh S.A., Goldberg J., Alkhafan F., Vitarello C., Rosenfield K., Lookstein R., Keeling B. (2024). Contemporary Management and Outcomes of Patients with High-Risk Pulmonary Embolism. J. Am. Coll. Cardiol..

[26] Stein P.D., Matta F., Lawrence F.R., Hughes M.J. (2018). Importance of Early Insertion of Inferior Vena Cava Filters in Unstable Patients with Acute Pulmonary Embolism. Am. J. Med..

[27] Stein P.D., Matta F., Hughes M.J. (2017). Inferior Vena Cava Filters in Elderly Patients with Stable Acute Pulmonary Embolism. Am. J. Med..

[28] Rousseau H., Del Giudice C., Sanchez O., Ferrari E., Sapoval M., Marek P., Delmas C., Zadro C., Revel-Mouroz P. (2021). Endovascular therapies for pulmonary embolism. Heliyon.

[29] Stein P.D., Matta F. (2012). Case fatality rate with pulmonary embolectomy for acute pulmonary embolism. Am. J. Med..

[30] Stein P.D., Matta F., Lawrence F.R., Hughes M.J. (2018). Usefulness of Inferior Vena Cava Filters in Unstable Patients with Acute Pulmonary Embolism and Patients Who Underwent Pulmonary Embolectomy. Am. J. Med..

[31] Stein P.D., Matta F., Hughes M.J. (2020). Effect on Mortality with Inferior Vena Cava Filters in Patients Undergoing Pulmonary Embolectomy. Am. J. Med..

[32] Davies M.G., Hart J.P. (2023). Current status of ECMO for massive pulmonary embolism. Front. Cardiovasc. Med..

[33] Liu Z., Chen J., Xu X., Lan F., He M., Shao C., Xu Y., Han P., Chen Y., Zhu Y. (2022). Extracorporeal Membrane Oxygenation-First Strategy for Acute Life-Threatening Pulmonary Embolism. Front. Cardiovasc. Med..

[34] Farkas J. Internet Book of Critical Care (IBCC). https://emcrit.org/ibcc/pe/#surgical_thrombectomy.

[35] Sherk W.M., Khaja M.S., Jo A., Marko X., Williams D.M. (2020). Bedside intravascular ultrasound-guided fibrin sheath balloon maceration and inferior vena cava filter placement during extracorporeal membranous oxygenation decannulation. J. Vasc. Surg. Cases Innov. Tech..

[36] Stein P.D., Matta F., Hughes M.J. (2018). Inferior Vena Cava Filters in Stable Patients with Acute Pulmonary Embolism Who Receive Thrombolytic Therapy. Am. J. Med..

[37] Stein P.D., Matta F., Hughes M.J. (2020). Effectiveness of Inferior Vena Cava Filters in Patients with Stable and Unstable Pulmonary Embolism and Trends in Their Use. Am. J. Med..

[38] Bikdeli B., Wang Y., Minges K.E., Desai N.R., Kim N., Desai M.M., Spertus J.A., Masoudi F.A., Nallamothu B.K., Goldhaber S.Z. (2016). Vena Caval Filter Utilization and Outcomes in Pulmonary Embolism: Medicare Hospitalizations from 1999 to 2010. J. Am. Coll. Cardiol..

[39] Stein P.D., Matta F., Hughes M.J. (2019). Inferior Vena Cava Filters in Stable Patients with Pulmonary Embolism and Heart Failure. Am. J. Cardiol..

[40] Stein P.D., Matta F., Lawrence F.R., Hughes M.J. (2018). Inferior Vena Cava Filters in Patients with Acute Pulmonary Embolism and Cancer. Am. J. Med..

[41] Mellado M., Pijoan J.I., Jiménez D., Muriel A., Aujesky D., Bertoletti L., Decousus H., Barrios D., Clará A., Yusen R.D. (2016). Outcomes Associated with Inferior Vena Cava Filters Among Patients with Thromboembolic Recurrence during Anticoagulant Therapy. JACC Cardiovasc. Interv..

[42] Stein P.D., Matta F., Lawrence F.R., Hughes M.J. (2019). Inferior Vena Cava Filters in Patients with Recurrent Pulmonary Embolism. Am. J. Med..

[43] Bajda J., Park A.N., Raj A., Raj R., Gorantla V.R. (2023). Inferior Vena Cava Filters and Complications: A Systematic Review. Cureus.

[44] Hohenwalter E.J., Stone J.R., O’Moore P.V., Smith S.J., Selby J.B., Lewandowski R.J., Samuels S., Kiproff P.M., Trost D.W., Madoff D.C. (2017). Multicenter Trial of the VenaTech Convertible Vena Cava Filter. J. Vasc. Interv. Radiol..

[45] Covello B., Radvany M. (2022). Back to the Basics: Inferior Vena Cava Filters. Semin. Interv. Radiol..

[46] De Gregorio M.A., Guirola J.A., Urbano J., Díaz-Lorenzo I., Muñoz J.J., Villacastin E., Lopez-Medina A., Figueredo A.L., Guerrero J., Sierre S. (2020). Spanish multicenter real—Life registry of retrievable vena cava filters (REFiVeC). CVIR Endovasc..

