# Supplementary material for: The Role of IVC Filters in the Management of Acute Pulmonary Embolism
Source: J Clin Med. 2024 Mar 5;13(5):1494. doi: 10.3390/jcm13051494 (PMC10935447; doi:10.3390/jcm13051494)

Supplementary Table S1. IVC-filter-related complications and definitions.

IVC-Filter-Related Complications	Definition	Reported Range of Frequency of Complications
IVC thrombotic occlusion	The detection of a thrombus obstructing the IVC following filter insertion can be confirmed through various diagnostic modalities, including ultrasound (US), computed tomography (CT), magnetic resonance (MR) imaging, venography, or autopsy.	2% to 30%
IVC penetration	The penetration of the vein wall by a filter strut or anchor device is identified by transmural incorporation extending more than 3 mm beyond the wall of the IVC. This penetration can be demonstrated through imaging techniques such as CT or venography, or it can be observed at autopsy.	Up to 41%
Filter embolization	Post-deployment movement of the filter or its components refers to the displacement of the filter or its parts to a distant anatomic site, completely out of the intended target zone.	Up to 11%
Filter movement	A change in the position of the filter, either cranial or caudal, is considered significant if it exceeds 2 cm from its originally deployed position. This change in position can be documented through various imaging methods such as plain radiography, CT, or venography.	Up to 18%
Filter fracture	Any compromise in the structural integrity of a filter, such as breakage or separation, can be documented through imaging studies or observation during autopsy.	2% to 10%
Insertion problems	Malfunctions of the filter or deployment system encompass various issues, including incomplete filter opening, filter tilt exceeding 15° from the IVC axis (e.g., non-self-centering filters), misplacement of the filter outside the infrarenal IVC when the intended placement is within the infrarenal IVC (e.g., when a portion of the filter is within one iliac vein), or prolapse of filter components. When filter malposition necessitates surgical or endovascular removal, it is categorized as an insertion complication.	5% to 23%
Access site thrombosis	The development of either occlusive or non-occlusive thrombus at the venotomy site subsequent to filter insertion can be verified through US or other imaging modalities.	3% to 10%
Access site complications with clinical sequelae	Complications related to IVC filter insertion encompass various issues, such as arteriovenous fistula, hematoma, or bleeding that necessitates a transfusion. These complications may result in hospitalization, either admission or an extended stay, and may require further medical treatment.	Up to 25%

Supplementary Figure S1. Radiographic images of IVC-filter-related complications

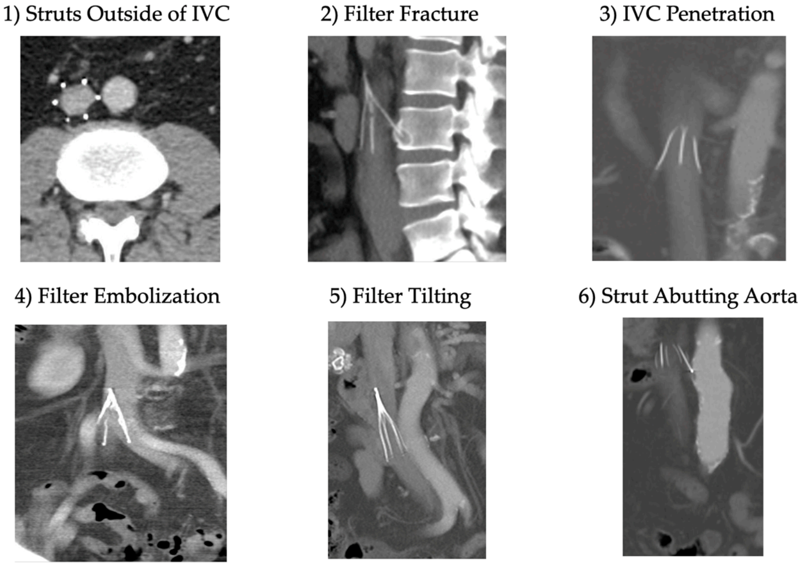

Supplement: Supplementary file 1 [file jcm-13-01494-s001.zip › jcm-2855183-supplementary.pdf]
